# Efficacy and safety of a bioadhesive gel containing propolis extract, nanovitamin C and nanovitamin E on desquamative gingivitis: a double-blind, randomized, clinical trial

**DOI:** 10.1007/s00784-022-04653-0

**Published:** 2022-07-28

**Authors:** José González-Serrano, Julia Serrano, Mariano Sanz, Jesús Torres, Gonzalo Hernández, Rosa María López-Pintor

**Affiliations:** 1 Department of Dental Clinical Specialties, ORALMED Research Group, School of Dentistry, Complutense University of Madrid. Plaza Ramón y Cajal S/N, 28040 Madrid, Spain; 2 Departamento de Especialidades Clínicas Odontológicas. Facultad de Odontología, Universidad Complutense de Madrid. Plaza Ramón Y Cajal S/N, 28040 Madrid, Spain

**Keywords:** Desquamative gingivitis, Oral lichen planus, Mucous membrane pemphigoid, Propolis, Antioxidants

## Abstract

**Objectives:**

To evaluate the efficacy of a gel-containing propolis extract, nanovitamin C, and nanovitamin E as adjuvants to professional plaque removal on desquamative gingivitis (DG).

**Materials and methods:**

A randomized clinical trial was conducted on patients suffering DG due to mucocutaneous diseases. Patients received professional supragingival prophylaxis with oral hygiene instructions and were randomly assigned to use test or control gels as toothpaste and to apply it on DG lesions 3 times/day for 4 weeks. DG clinical score (DGCS), clinical periodontal variables, and visual analog scale (VAS) for pain and oral health impact profile (OHIP-14) were collected at baseline, 2 and 4 weeks.

**Results:**

Twenty-two patients were randomly assigned to test (*n* = 11) or control group (*n* = 11). Eighteen had diagnosis of oral lichen planus and four of mucous membrane pemphigoid. DGCS statistically decreased in both groups after treatment with no significant differences between groups. Clinical periodontal outcomes decreased in both groups, but no significant differences were observed. Periodontal variables statistically improved only in test group after treatment. VAS and OHIP-14 scores decreased in test and control groups without significant differences. However, only one test group showed a statistically significant decrease in VAS and OHIP-14 scores after treatment. No adverse effects were reported.

**Conclusions:**

Test gel may alleviate DG and improve quality of life without side effects.

**Clinical relevance:**

A gel-containing propolis extract, nanovitamin C, and nanovitamin E as adjuvants to mechanical debridement may improve both clinical and patient related outcomes in DG patients without side effects.

Clinical trial registration.

The study protocol was registered at clinicaltrials.gov with the following number: NCT05124366 on October 16, 2021.

## Introduction

Desquamative gingivitis (DG) is a clinical entity characterized by epithelial desquamation associated to erythema, erosions, and ulcerations of the marginal and/or attached gingiva frequently associated to pain and discomfort [1]. DG can vary in extent, affecting in many cases only the anterior vestibular sector, but it can also affect the gingiva in a generalized manner. The severity of the cases is also variable. It can be mild; in these cases, patients may present erythematous and edematous gingival lesions. However, in other cases, the severity may be greater presenting areas with desquamation, blisters, pseudomembranes, erosions, ulcerations, and the possible presence of spontaneous hemorrhage. Mild forms are associated with discomfort with the ingestion of spicy and acidic foods, or also with the use of certain toothpastes and mouthwashes. However, severe forms usually present with spontaneous pain that makes eating and oral hygiene difficult, thus worsening the quality of life of patients [1–3].

Several mucocutaneous diseases have been associated to DG being oral lichen planus (OLP) and mucous membrane pemphigoid (MMP) the most frequent, although others as pemphigus vulgaris, lupus erythematosus, erythema multiforme, graft-versus-host disease, or epidermolysis bullosa have also been related [2–4]. DG has been recently categorized as a type of gingival disease not induced by dental plaque in the new classification of periodontal and peri-implant diseases [5, 6]. However, patients with chronic DG usually have poor oral hygiene due to their discomfort and frequent bleeding when brushing, what leads to plaque-induced gingivitis, what usually increases the severity of DG, leading to more pain and a greater impact on the patient’s quality of life [7–11]. This fact has been corroborated by some studies reporting improvement in clinical manifestations of DG and patient’s oral health-related quality of life (OHRQoL) when implementing effective dental plaque control measures [8, 12–16].

OLP, the most frequently associated disease with DG, is a chronic inflammatory autoimmune disease, in which, proinflammatory cytokines (TNF-α, IL1, IL6, CRP) have an important pathogenic role. In fact, elevated concentrations of these cytokines in saliva and serum have been significantly associated in these patients with basement membrane degeneration and progression of the lesions [17–20]. Similarly, increases in the expression of matrix metalloproteinases (MMP), such as MMP-1 and MMP-9 have been found in the gingival tissues of OLP patients [15, 21]. Moreover, IL-17 is elevated in the serum of patients with autoimmune-blistering diseases compared to controls, which promotes the release of IL-6, IL-8, TNF-α, MMP-9, or MMP-13 [22].

Due to the presence of this chronic inflammation, meticulous plaque removal does not usually result in complete resolution of DG [14, 23], and other specific treatments, such as topical corticosteroids are needed to reduce the DG lesions and their associated signs and symptoms [24–27]. Other anti-inflammatory and immunosuppressant treatments such as systemic steroids, topical tacrolimus, topical pimecrolimus, mycophenolate, methotrexate, dapsone, and retinoic acid have also been used in refractory cases or in presence of extensive lesions of DG [28]. However, the long-term use of these drug regimes is usually associated to of the advent of side effects such as oral candidiasis, hyperglycemia, hypertension, osteoporosis, or Cushing’s syndrome [26]. Therefore, alternative long-term therapies have been explored, mainly including natural products such as aloe vera, curcumin, honey, or micronutrients [26, 29].

A recent meta-analysis has shown that propolis may have beneficial effects in patients, since propolis extracts significantly reduce serum TNF-α and CRP concentrations [30] and have an inhibitory effect in MMP-9 activity [31, 32]. Recently, a formulation as a gingival bioadhesive gel-containing propolis extract, nanovitamin C, and nanovitamin E has demonstrated clinical improvements when treating peri-implant mucositis [33], in the pain reduction after the surgical extraction of impacted lower third molars [34], and in the treatment of oral erosive lesions of OLP [35]. It was, therefore, the objective of this randomized clinical trial to evaluate the efficacy and safety of the use of this bioadhesive gel-containing propolis extract, nanovitamin C, and E as an adjuvant to professional plaque removal in the treatment of DG.

## Material and methods

### Study design

This clinical study was designed as a randomized clinical trial (RCT) following the CONSORT guidelines for reporting (http://www.consort-statement.org/). The study protocol was also registered at clinicaltrials.gov (NCT05124366) and was approved by the Ethics Committee at Hospital Clínico San Carlos, (Madrid, Spain) (19/345-R_X Tesis). The study was conducted according to the principles of the Declaration of Helsinki on clinical studies with humans.

### Patients

Patients were selected from those attending the Postgraduate Clinic of Oral Medicine at the Faculty of Odontology in the University Complutense of Madrid (Spain) between September 2019 and December 2021. Two specialists in Oral Medicine (JG-S and RML-P) screened the patients with a comprehensive oral examination and if fulfilling a pre-defined set of inclusion and exclusion criteria they were entered in the study. Those selected patients were informed on the specifics of the clinical trial and if agreed to participate they signed the informed consent approved by the Ethical Committee.

The criteria for inclusion were patients (1) over 18 years of age with a clinical diagnosis of DG (erythema, epithelial desquamation, atrophy, painful erosions, or ulceration of the free and/or attached gingiva); (2) with clinical and histological diagnosis of OLP according to the 2016 American Academy of Oral and Maxillofacial Pathology criteria [36] or MMP according to the 2015 World Workshop on Oral Medicine criteria [37]; (3) clinical diagnosis of plaque-induced gingivitis or patients in periodontal maintenance with probing depths (PD) ≤ 5 mm.

The exclusion criteria were patients with (1) diagnosis of systemic diseases or conditions that could alter the results of the study (uncontrolled diabetes mellitus, immunosuppression, infectious diseases, rheumatoid disease, history of bisphosphonate treatment, radiotherapy, chemotherapy, immunotherapy); (2) active medication with drugs associated with gingival enlargement such as cyclosporine, calcium channel blockers, and phenytoin; (3) previous treatments with topical corticosteroids within the past 4 weeks or 8 weeks with systemic corticosteroids; (4) previous treatments with local and/or systemic antibiotics and/or anti-inflammatories within the last 3 months; (5) pregnancy or breastfeeding; (6) being smoker; (7) using anti-plaque or anti-gingivitis mouth rinses; (8) with a history of allergy to any component of the tested gel.

### Randomization and treatment allocation

At the baseline visit, patients had a clinical examination where DG clinical score (DGCS), clinical periodontal variables, and patient-reported outcomes were collected. Then, all patients received a professional supragingival prophylaxis with an ultrasonic device (Piezon Master, EMS, Nyon, Switzerland) and standardized oral hygiene instructions by one of the clinical investigators (JG-S). At this visit, each patient was assigned a number according to the order of entry into the study, and test and control treatments were randomly assigned using a computer-generated list. Allocation to these treatments were carried out by the study monitor, not involved in the clinical aspects of the trial using closed opaque bags numbered from 1 to 22. Patients and clinicians were blinded to the treatment assignment. Randomization codes were not revealed until the trial was finished.

### Treatments

Test (NBF gingival gel, Sungwon pharmaceutical co, Goyang, South Korea) and control gels were prepared in identical tubes of 30 g presenting the same color, flavor, and density. Both gels contained the following components: sodium-monofluorophosphate, silicon dioxide, glycerin, D-sorbitol, polyethylene glycol, sodium carboxymethylcellulose, xylitol, sterol glycoside, peppermint oil (0.13%), L-menthol (0.4%), methyl hydroxybenzoate, and deionized water. Only the test gel contained 2% propolis extract (collected in autumn from September to November in the South-East of South Korea), 0.2% ascorbic acid, and 0.2% tocopherol acetate. E155/151 coloring was added in the control gel to simulate the brown color of the propolis. The details on the pharmacodynamics of the tested gel, including the cumulative release of the propolis extract and the chromatographic results were reported in a previous publication from our research group [33].

Each patient received 3 tubes of 30 g of test gel or control gel. The patients also received a soft toothbrush (CS5460 Curaprox, Curaden AG, Kriens, Switzerland) and were instructed to carefully apply their assigned gel as a toothpaste 3 times a day for 4 weeks using the modified Bass brushing technique. Also, when appropriate, the patients were instructed on the use of interdental brushes (Interprox Plus, Dentaid, Barcelona, Spain). Moreover, the patients were instructed to apply the gel with clean hands on the DG lesions, and prompted not to eat, drink, rinse, or use any other oral treatment for 30 min after the application.

### Clinical outcomes

The primary outcome was the evaluation of the efficacy of the tested interventions on the extent and severity of the desquamative gingivitis lesions. We used the DGCS as described by Arduino et al. 2017 [38], which uses the following scoring criteria: (0) no detectable gingival lesions present; (1) only white lesions; (2) mild erythema (< 3 mm from the gingival margins); (3) one or more blister or clinically obvious erythema (> 3 mm from the gingival margins); (4) erosion or ulcer. The evaluation should be carried out both in the buccal as well as in the lingual/palatal aspect, collecting a value for each sextant (12 in total, with a maximum score of 48 and a minimum score of 0). The visual examination was carried out by single calibrated and blinded examiner (RML-P) who had been previously calibrated by evaluating 6 patients with DG using consecutive DGCS scores within 60 min, achieving an intra-examiner reproducibility of 91%. The examinations were performed with good lighting conditions using a calibrated probe (PCPUNC15; Hu-Friedy, Chicago, IL, USA) to measure the size of the lesions.

As secondary outcomes, we assessed the following clinical periodontal outcome variables measured in all teeth/implant at six sites per tooth/implant present, using the same calibrated probe with a force of 0.2 N: (a) probing depth (PD); (b) bleeding on probing (BOP), and (c) plaque index (PI) using a disclosing dye.

DGCS and periodontal outcome variables were registered at baseline, 2 and 4 weeks since starting the tested interventions.

### Patient-reported outcome measures (PROMs): pain and OHRQoL

The perception of pain was recorded by the patient using a visual analog scale (VAS) of 10 cm. OHRQoL was assessed using the Spanish version of the Oral Health Impact Profile (OHIP-14) questionnaire [39], which quantifies the impact of the treatment in the patient’s oral health-related quality of life. Both PROMs variables were collected at baseline and 2 and 4 weeks after starting the tested interventions.

### Compliance

All participants were asked to bring the gel tubes at the 1-month follow-up visit to measure the remaining weight of the gel with a calibrated digital scale.

### Sample size calculation and statistical analysis

The sample size calculation was based on an estimation of mean differences (MD) of 4 between test and control in reduction of DGCS, with a standard deviation (SD) of 2.3 [14], an alpha-risk of 5%, and a statistical power of 95%. With this estimation, sample size resulted in 9 patients in each group, which after assuming a potential drop-out rate of 15%, we determined a sample size of 10 participants per group.

Changes in the primary, secondary, and PROMs outcome variables were calculated between baseline—2 weeks, 2–4 weeks, and baseline—4 weeks. Inter-group differences in categorical variables were determined using Chi-square test or Fisher’s exact test. Shapiro–Wilk test was used to determine the normality of the distribution of the quantitative variables. Inter-group differences were determined by Mann–Whitney *U* test. Friedman’s test with Bonferroni correction was used to evaluate intragroup differences in the clinical outcome variables over time. Differences were considered significant if *p* was ≤ 0.05. The statistical analysis was done using SPSS version 27.0 (SPSS Inc. New York, NY, USA).

## Results

### Study sample

Twenty-eight subjects with DG were screened for inclusion, but 4 did not meet the inclusion criteria and 2 refused to participate, leading to a final sample of 22 patients that were randomized to participate in the RCT.

**Fig. 1:**
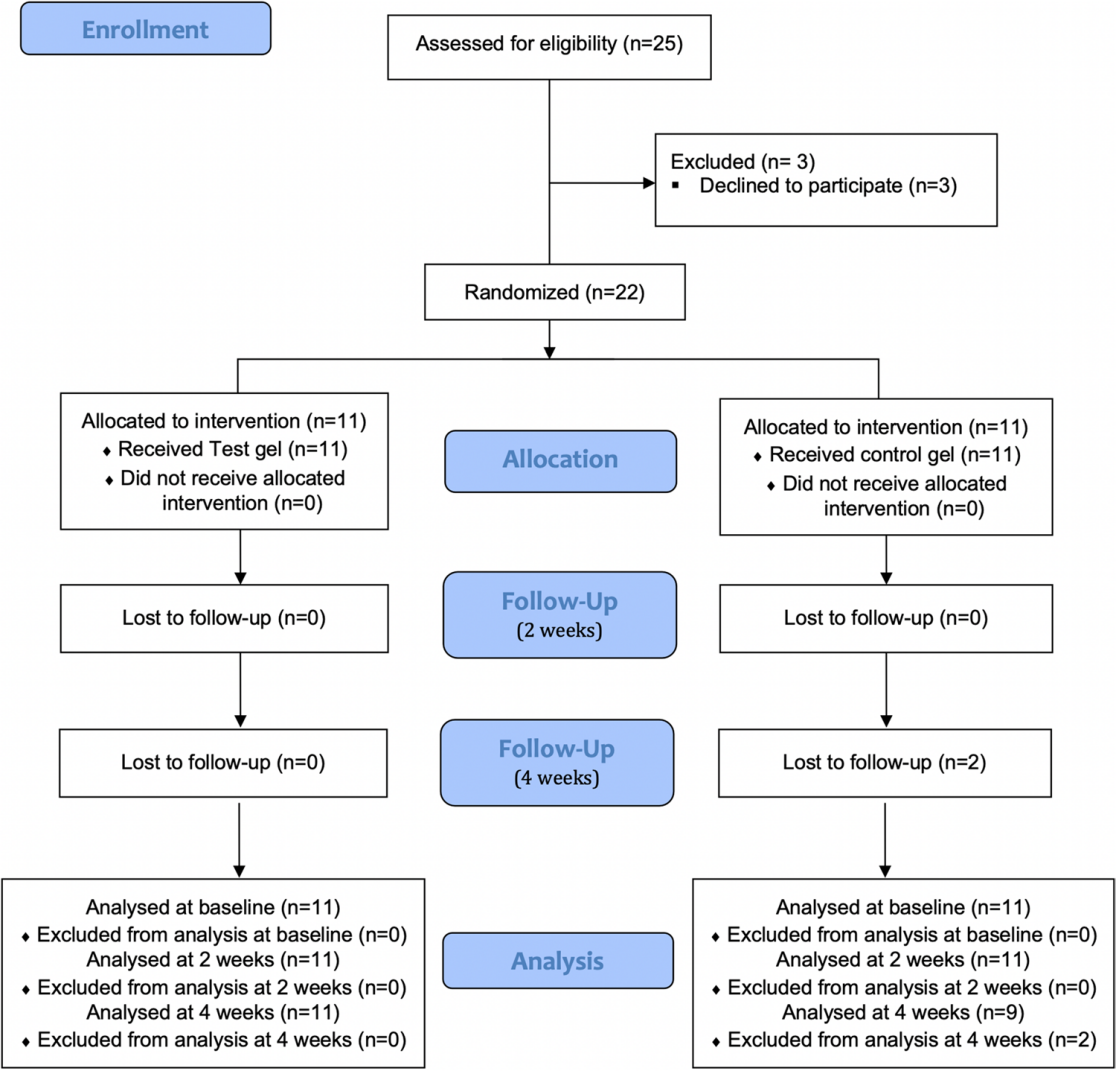
Consort flow diagram on subject enrollment, allocation, follow-up, and analysis

All the patients were female, having 18 a diagnosis of OLP and 4 a diagnosis of of MMP. Eleven patients were randomly assigned to the test group (mean age: 62.82 ± 13.86) and 11 to the control group (mean age: 68.18 ± 12.98). Two patients from the control group dropped out by not attending the 4-week follow-up visit. Hence, 22 patients completed the 2-week visit and 20 patients completed the 4-week evaluation visit (Fig. 1). Table 1 depicts the descriptive baseline information of this sample population. There were no statistically significant differences in any of these baseline characteristics when comparing the test and control groups.

**Table 1:** Characteristics of the patients included

	Test group (*n* = 11)	Control group (*n* = 11)	*P*-value test vs. control group
Age (years)	62.82 ± 13.86	68.18 ± 12.98	*p* = 0.4
Gender: female/male	11/0	11/0	*p* = 1
Oral diseases associated to DG	OLP^a^ (*n* = 9) MMP^b^ (*n* = 2)	OLP (*n* = 9) MMP (*n* = 2)	*p* = 1
Time since diagnosis (months)	52.82 ± 50.13	51.09 ± 48.66	*p* = 0.85

^a^*OLP*, oral lichen planus; ^*b*^*MMP,* mucous membrane pemphigoid

### Clinical outcomes

Table 2 depicts the mean DGCS values at baseline, 2-week and 4-week visits, as well as the mean changes in periodontal outcome variables. Four weeks after treatment complete DG resolution was found in 2 patients (18.18%) of the test group, while none in the control group. DGCS scores significantly decreased in both groups after treatment (test group *p* = 0.0001; control group *p* = 0.001), but there were no statistically significant differences between groups (*p* = 0.37).

**Table 2:** Mean values of clinical results at baseline, 2-week, and 4-week follow-ups. Mean changes in clinical results between baseline and 4-weeks follow-up

		Baseline			*p* value	2 weeks			4 weeks			Baseline 2 weeks			*p value*	2 weeks–4 weeks			*p* value	Baseline 4 weeks			*p* value	*p* intragroup Baseline 2 weeks 4 weeks
	Group	*N*	Mean	SD^e^		*N*	Mean	SD	*N*	Mean	SD	*N*	Mean	SD		*N*	Mean	SD		*N*	Mean	SD		
**DGCS**^a^	Test	11	14.82	9.36	0.56	11	9.18	9.17	11	8.27	9.08	11	5.64	2.87	0.27	11	0.91	3.39	0.88	11	6.55	5.11	0.37	0.0001*
	Control	11	15.91	5.52		11	12.73	4.76	9	11	5.41	11	3.18	4.85		9	2.22	4.47		9	5.22	3.73		0.001*
**PD**^b^ **(mm)**	Test	11	2.82	0.34	0.95	11	2.47	0.36	11	2.55	0.19	11	0.35	0.28	0.15	11	-0.08	0.24	0.5	11	0.27	0.23	0.46	0.006*
	Control	11	2.9	0.53		11	2.71	0.43	9	2.75	0.6	11	0.19	0.25		9	-0.01	0.35		9	0.19	0.42		0.46
**BOP**^**c**^** (%)**	Test	11	68.45	14.26	0.9	11	55.36	19.09	11	53.18	11.07	11	13.09	6.39	0.7	11	2.18	14.65	0.71	11	15.27	11.73	0.88	0.002*
	Control	11	68.18	17.06		11	55.91	19.18	9	49.89	22.87	11	12.27	19.12		9	6.11	11.27		9	16.44	18.13		0.07
**PI**^d^ **(%)**	Test	11	78.64	16.59	0.37	11	56.73	23.58	11	54.36	21.19	11	21.91	17.94	0.22	11	2.36	17.28	0.88	11	24.27	19.01	0.33	0.002*
	Control	11	81.09	19.36		11	68.91	24.23	9	67.89	17.6	11	12.18	21.73		9	1.78	14.85		9	12.55	17.53		0.03*

^a^*DGCS*, desquamative gingivitis clinical score; ^b^*PD*, probing depth; ^c^*BOP*, bleeding on probing; ^d^*PI*, plaque index; ^e^*SD*, standard deviation; ^*^statistically significant results

Clinical periodontal outcomes improved in both groups, but not statistically significant differences were observed between groups. Mean differences in PD, BOP, and PI significantly improved between baseline and 4 weeks in test group after treatment (*p* = 0.006; *p* = 0.002; *p* = 0.002, respectively). Only PI significantly decreased in control group from baseline to a 4-week’s visit (*p* = 0.03).

### PROMs: pain and OHRQoL

The VAS scores decreased in test and control groups, without significant differences between groups (*p* = 0.23). However, in the test group, VAS scores for pain significantly decreased after treatment (*p* = 0.001). The total OHIP-14 scores decreased in test and control groups, without statistically significant differences between groups (*p* = 0.37). Only the test group showed a significant decrease in total OHIP-14 scores after treatment (*p* = 0.002). The components of the OHRQol that improved the most in the test group were “physical pain” (*p* = 0.001), “psychological disability” (*p* = 0.009), and “social disability” (*p* = 0.003). Only the component “handicap” demonstrated statistically significant reductions in the control group after treatment (*p* = 0.03). See Table 3.

**Table 3:** Mean values of clinical results at baseline, 2-week and 4-week follow-ups. Mean changes in clinical results between baseline and 4-weeks follow-up

		Baseline			*p* value	2 weeks			4 weeks			Baseline 2 weeks			*p value*	2 weeks to 4 weeks			*p* value	Baseline 4 weeks			*p* value	*p* intragroup Baseline 2 weeks 4 weeks
	Group	*N*	Mean	SD		*N*	Mean	SD	*N*	Mean	SD	*N*	Mean	SD		*N*	Mean	SD		*N*	Mean	SD		
VAS	Test	11	4.64	1.96	0.80	11	2.27	1.27	11	1.91	2.02	11	2.36	1.69	0.12	11	0.36	2.29	0.77	11	2.73	2.49	0.23	0.001*
	Control	11	4.27	1.9		11	3.18	1.72	9	3	2.35	11	1.09	1.38		9	0.56	2.35		9	1.33	2.74		0.06
OHIP-14																								
Functional limitation	Test	11	0.91	1.87	0.27	11	0.82	0.98	11	0.64	1.5	11	0.09	1.58	0.3	11	0.18	1.33	0.77	11	0.27	0.47	0.82	0.31
	Control	11	1.27	1.35		11	0.72	1.27	9	0.67	1	11	0.54	0.93		9	0.11	0.6		9	0.33	0.5		0.17
Physical pain	Test	11	4.73	2.05	0.95	11	3.18	2.14	11	2.55	2.11	11	1.55	1.75	0.37	11	0.64	2.25	0.3	11	2.18	2.4	0.13	0.001*
	Control	11	4.82	2.4		11	4.09	2.39	9	3.67	2.18	11	0.73	0.79		9	0	1.73		9	0.78	1.72		0.12
Psychological discomfort	Test	11	6.27	2.20	0.08	11	5	2.76	11	4.18	3.03	11	1.27	2.28	0.75	11	0.82	2.4	0.77	11	2.09	2.47	0.15	0.07
	Control	11	4.55	2.38		11	4.18	2.14	9	4.11	2.2	11	0.36	2.11		9	0.11	1.45		9	0.11	1.96		0.42
Physical disability	Test	11	2	2.79	0.17	11	1.64	2.16	11	1.18	1.89	11	0.36	1.91	0.7	11	0.45	0.82	0.77	11	0.82	1.54	0.94	0.26
	Control	11	2.91	1.97		11	2.55	2.62	9	2.44	1.59	11	0.36	1.5		9	0.11	2.03		9	0.56	1.01		0.37
Psychological disability	Test	11	2.73	2.28	0.75	11	1.45	1.81	11	1	1.84	11	1.27	1.49	0.08	11	0.45	0.82	1	11	1.73	1.42	0.13	0.003*
	Control	11	2.55	2.38		11	2.18	1.72	9	1.44	1.74	11	0.36	1.03		9	0.78	1.72		9	1.11	2.67		0.23
Social disability	Test	11	1.55	1.92	0.95	11	0.91	1.81	11	0.73	1.79	11	0.64	0.81	0.56	11	0.18	0.6	0.71	11	0.82	1.08	0.66	0.009*
	Control	11	1.27	1.27		11	0.82	1.08	9	0.78	0.97	11	0.45	0.82		9	0.22	0.44		9	0.55	0.88		0.08
Handicap	Test	11	1.64	2.25	0.52	11	1.09	2.12	11	1.27	1.9	11	0.55	0.82	0.75	11	-0.18	1.17	0.26	11	0.36	1.69	0.55	0.24
	Control	11	2	2		11	1.18	1.08	9	0.89	0.78	11	0.82	1.25		9	0.33	0.5		9	0.78	1.09		0.03*
Total OHIP-14 score	Test	11	19.82	10.66	0.9	11	14.09	10.12	11	11.55	10.19	11	5.73	7.27	0.65	11	2.54	4.87	0.77	11	8.27	7.9	0.37	0.002*
	Control	11	19.36	10.88		11	15.73	8.06	9	14	6.98	11	3.64	4.54		9	1.67	4.39		9	4.22	5.49		0.18

^*^Statistically significant results

### Patient compliance and adverse effects

At 1 month, both groups demonstrated a similar degree of compliance with no statistical differences, as measured by the remaining weight of the gel tubes returned (mean in grams of the remaining gel tubes: test 9.84 ± 2.69; control: 9.86 ± 3.48, *p* = 0.88). Adverse reactions or discomfort with the use of the test or control gels were not reported by any patient.

## Discussion

The results from this RCT showed that a gel-containing propolis extract, nanovitamins C, and nanovitamins E when used topically as an adjuvant to the professional mechanical debridement may decrease the extent and severity of DG lesions, as measured by the DGCS scoring system after treatment. Although this reduction was of a higher magnitude in the test when compared with the control group, differences between the groups were not statistically significant 4 weeks after the initiation of the tested therapy. Similarly, PD, BOP, PI, VAS for pain and total OHIP-14 scores significantly improved in the patients from the test group, but differences between groups were not statistically significant at the end of the evaluation period.

The tested gel has already shown antibacterial and anti-inflammatory effects in the tissues surrounding dental implants [33], as well as to relieve pain after the extraction of impacted third molars [34], which may be due to the regenerative and antioxidant effect of propolis and nanovitamins C and E present in the gel. Other studies have also attributed micronutrients such as vitamin C and E, a preventive role in periodontal diseases [40], and have shown to reduce gingival inflammation in periodontitis patients when supplemented to standard non-surgical periodontal therapy [41]. Also, propolis has been reported as effective as 0.1% triamcinolone acetonide in the reduction of pain and erythema scores in patients with symptomatic OLP after 2 weeks of therapy [42]. Moreover, the tested gel did not contain sodium lauryl sulfate, which is related to desquamation of the oral mucosa, and therefore it is not recommended in these patients [43]. For all these reasons, the study gel may be an alternative in patients with DG.

There is only weak evidence for the superiority of any intervention over placebo in the treatment of DG [44]. The reduction in DG lesions after improvement in oral hygiene and plaque control have been reported in different studies. Among these studies, there are differences in methodology such as the number of plaque removal sessions, the treatment (supra and/or subgingival), the number of oral hygiene instruction sessions, and the toothbrush to be used (electric or manual) [16]. These studies have shown an improvement in DG-associated symptomatology, periodontal variables, mucosal disease score, and/or clinical indices [8, 13–15]. Some of these studies had no control group [8], or their control group was periodontally healthy [15]. There is another study that achieved improvement in quality of life and clinical severity of DG, but they used corticosteroids along with biofilm removal in some patients during the study [45].

There are also studies on the use of topical corticosteroids for GD [24, 25]. One of them did not have a control group [24], and the one that did have, did not obtain significant results between groups [25]. Furthermore, in the latter study, two patients treated with corticosteroids presented oral candidiasis. There is also a study that used oral sulfamethoxypyridazine, a long-acting sulfonamide antibiotic, for the treatment of DG secondary to MMP that had not responded to topical corticosteroids [46]. In this study, a significant improvement was observed after treatment, but it also had no control group and some patients had to drop out of the study due to significant side effects [46]. Therefore, there are few RCTs on the treatment of DG, and those that exist did not obtain significant results and, in some cases, showed adverse effects. In the present study, we have compared the improvement of DG after the use of a gel-containing propolis extract, nanovitamin C, and nanovitamin E or a control gel together with plaque control in all patients. No patient received any other treatment for DG or plaque control during the study. In addition, all patients suffered from DG and the groups were similar in age, gender, and diseases associated with DG. Therefore, the methodological design of this study is superior to some previous studies.

There is no consensus on the outcomes to be studied in clinical studies on the treatment of DG due to mucocutaneous diseases [8, 14, 45]. In fact, there is also no standardized index to measure treatment improvement [38, 47]. Although a mucosal index for OLP has been described [8, 47], a reproducible gingival clinical score for DG was lacking, which was an impediment to conducting quality studies [38]. To the best of our knowledge, there are only two studies evaluating DGCS [14, 15]. However, the present study is the first one using DGCS that compares a topical treatment versus a control group. DGCS changes obtained in test group at 1 month in the present study were greater than those obtained by Romano et al. [15] and Bianco et al. [14] studies. This may be due to the tested gel, since in previous studies only plaque control was applied.

In addition, in the present study, VAS for pain and OHIP-14 were collected. There are current studies [48] that have noted that to assess the efficacy of an OLP intervention it is important that VAS and total OHIP-14 scores are not ≤ 2.8 and ≤ 18, respectively. In the present study, although we included patients with OLP and MMP, our baseline scores regarding VAS and total OHIP-14 are higher in both groups than the scores indicated above. This is not the case in other studies for the DG treatment for VAS scores [45], nor for OHIP-14 total scores [14, 15, 45]. Furthermore, the VAS score in the present study was 1.91 at 1 month, which is an acceptable symptomatic state, and 3 in the control group, which already exceeds the borderline acceptable state [48].

Regarding the periodontal variables, the baseline PD of the present study are like the obtained in Romano et al. [15] and Bianco et al. [14] studies. We also observe how PD diminished in a similar manner to these studies [14, 15]. Regarding PI and BOP, the reductions obtained at 1 month in the present study are inferior to the obtained by Bianco et al. [14], which also performed professional plaque removal at week 2, 3, and 4 after therapy. In the present study, professional plaque removal was only done once before starting the treatment, but significant reductions for BOP and PI were obtained in the test group after treatment, which may indicate the anti-plaque and anti-inflammatory effect of the tested gel. For these reasons, and because there were statistically significant intragroup differences in DGCS, PD, BOP, PI, total OHIP-14, and VAS scores in the study group without side effects after treatment, there is a trend in favor of the tested gel, which may improve clinical findings, pain, and quality of life in patients with DG. In addition, the price of the study gel is similar to a topical corticosteroid preparation.

This RCT shares the strengths of this experimental design for evaluating the proposed intervention (a gel-containing propolis extract, nanovitamins C, and nanovitamin E as an adjuvant to professional mechanical debridement). It, however, has limitations, mainly the possible underpowered sample size and the insufficient follow-up, limited to 1 month. The limitations in sample size are frequent in the evaluation of interventions to treat mucocutaneous diseases due to the difficulty of finding patients who met all the inclusion and exclusion criteria. Furthermore, the positive impact of the professional plaque debridement and oral hygiene improvements in both patient’s groups may have disguised the possible differential effect of the tested gel. Regarding the limited follow-up, it was the objective of this study to evaluate the short-term efficacy of the tested gel and the obtained results, albeit not statistically significant, will aid us to properly design a future long-term RCT.

## Conclusions

In conclusion, the present study has shown improvements in the resolution of the DG lesions, the periodontal clinical outcomes and patient-reported outcomes when using a gel-containing propolis and nanovitamins C and E in conjunction with professional and personal plaque control, without reporting any adverse effects. Therefore, we suggest that this treatment may be an alternative therapy for the management of DG. However, more clinical trials with a larger sample and longer follow-up are needed to ascertain the real efficacy of this gel.
